# Supplementary material for: Transcriptomic Insights into Mechanisms of Early Seed Maturation in the Garden Pea (Pisum sativum L.)
Source: Cells. 2020 Mar 23;9(3):779. doi: 10.3390/cells9030779 (PMC7140803; doi:10.3390/cells9030779)

# Zhewan−1,10(3)

## Global Summary

%DE = 0.25

# genes with fdr < 0.2 = 452 ( 209 + / 243 −)

# genes with fdr < 0.1 = 420 ( 195 + / 225 −)

# genes with fdr < 0.05 = 397 ( 189 + / 208 −)

# genes with fdr < 0.01 = 322 ( 152 + / 170 −)

# genes in genesets = 0

<FC> = 0

<t−score> = 0

<p−value> = 0.01

<fdr> = 0.76

## Global Genelist

| Rank | ID | log(FC) | p−value | fdr | Metagene | Description |
|---|---|---|---|---|---|---|
| 1 | TRINITY_DN1 | −0.56 | 2e−16 | 3e−15 | 30 x 1 | |
| 2 | TRINITY_DN1 | 0.87 | 2e−16 | 3e−15 | 1 x 3 | |
| 3 | TRINITY_DN9 | −0.6 | 2e−16 | 3e−15 | 30 x 30 | |
| 4 | TRINITY_DN7 | −1.44 | 2e−16 | 3e−15 | 30 x 1 | |
| 5 | TRINITY_DN7 | 0.98 | 2e−16 | 3e−15 | 30 x 30 | |
| 6 | TRINITY_DN5 | −2.31 | 2e−16 | 3e−15 | 30 x 30 | |
| 7 | TRINITY_DN5 | 2.05 | 2e−16 | 3e−15 | 1 x 1 | |
| 8 | TRINITY_DN5 | 1.4 | 2e−16 | 3e−15 | 1 x 30 | |
| 9 | TRINITY_DN7 | −0.65 | 2e−16 | 3e−15 | 30 x 30 | |
| 10 | TRINITY_DN7 | 1.32 | 2e−16 | 3e−15 | 1 x 1 | |
| 11 | TRINITY_DN9 | −0.54 | 2e−16 | 3e−15 | 30 x 1 | |
| 12 | TRINITY_DN9 | −0.53 | 2e−16 | 3e−15 | 30 x 30 | |
| 13 | TRINITY_DN9 | −1.08 | 2e−16 | 3e−15 | 30 x 30 | |
| 14 | TRINITY_DN9 | −0.5 | 2e−16 | 3e−15 | 30 x 1 | |
| 15 | TRINITY_DN2 | 0.67 | 2e−16 | 3e−15 | 1 x 30 | |
| 16 | TRINITY_DN2 | 0.66 | 2e−16 | 3e−15 | 1 x 1 | |
| 17 | TRINITY_DN2 | −0.55 | 2e−16 | 3e−15 | 30 x 1 | |
| 18 | TRINITY_DN2 | −1.25 | 2e−16 | 3e−15 | 30 x 30 | |
| 19 | TRINITY_DN2 | 1.29 | 2e−16 | 3e−15 | 1 x 30 | |
| 20 | TRINITY_DN2 | 1.33 | 2e−16 | 3e−15 | 1 x 1 | |

**Portrait**

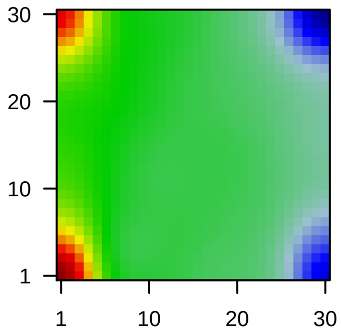


**Top 100 DE genes**

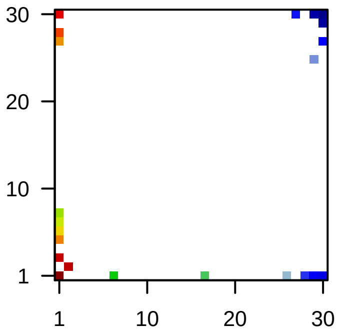


**p−values**

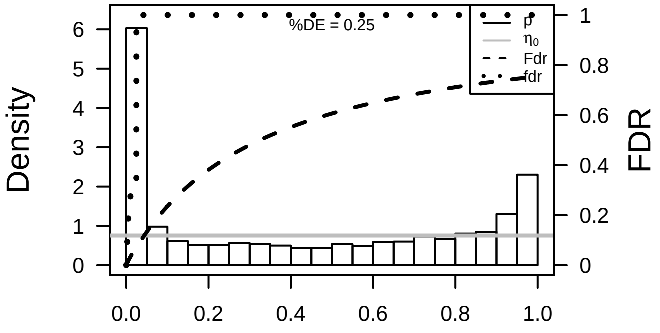

Supplement: Supplementary file 1 [file cells-09-00779-s001.zip › Supplementary materials/FileS4.zip!/FileS3/TFs - Results/Summary Sheets - Samples/Zhewan.1.10.3..pdf]
